# Patient removals: time to rethink exclusion in general practice?

**DOI:** 10.3399/BJGP.2025.0751

**Published:** 2025-12-16

**Authors:** Natassia Brenman, Francesca H Dakin, Jessica Drinkwater, Anne-Laure Donskoy, Kelly Howells, Clive Rowe, Sara E Shaw, Jackie van Dael

**Affiliations:** 1 Nuffield Department of Primary Care Health Sciences, University of Oxford, Oxford, UK; 2 Centre for Primary Care and Health Services Research, University of Manchester, Manchester, UK; 3 School of Sociology, Politics and International Studies, University of Bristol, Bristol, UK; 4 NIHR School for Primary Care Research, University of Manchester, Manchester, UK; 5 Chair of the Patients Participation Group (PPG) of the Adelaide, and St Levan's Doctors Surgeries, UK

On 24 July 2025, *Pulse* reported the death of a Devonshire man who had been removed from mainstream general practice.^1^ Although no further details were given, the coroner’s investigation has suggested that the man, diagnosed with autism spectrum disorder, had been receiving insufficient care through the ‘Special Allocation Scheme’ (SAS). Previously known as the ‘violent patient service’, the SAS is a national (England-wide) service for patients who have been removed from their regular GP provider after being reported for violence or aggression. It was developed in an attempt to maintain the safety of GP staff, while providing continued primary care to patients in a secure setting.^2^ Since the introduction of the scheme in 2004, levels of violence and aggression in general practice have been escalating and remain a pressing concern.^3^

While the SAS has received little attention in the wider media and research, the patient’s death has prompted calls for tighter protocols and safeguarding measures when general practices consider referring patients to the SAS. In July 2024, NHS England updated its guidance recommending that general practices consider disabilities, neurodiversity, and safeguarding before making such referrals.^1,2^ However, questions remain about when SAS registration is the appropriate course of action, if the service itself is unsuitable for many patients with complex needs.

## Who is the Special Allocation Scheme for?

The Devonshire incident highlights a tension at the heart of SAS service delivery: patients placed on the scheme are often those who would most benefit from trusting relationships developed through continuity of care, and access to a local GP and other primary care services (for example, social prescribing, mental health support, and drug and alcohol services). However, SAS services are de facto temporary and almost always located outside the patients’ local area. While SAS-registered patients have a diverse range of needs, many experience severe and multiple disadvantage (SMD), which is associated with experiences of trauma and complex mental health.^4^ Existing evidence demonstrates that, despite their need, people with SMD experience significant barriers to accessing primary care, stigma, and discrimination, and (for some) a low uptake of specialist services.^5^ Removing these patients from their usual GP surgery risks exacerbating these challenges, prompting calls from the third sector to introduce more trauma-informed practice to SAS provision.^6^ Given the high stakes of patient removals, the updated guidance for SAS referrals is important; additional considerations are needed to avoid inappropriate referrals. However, there is still a risk that patients may fall through the cracks of primary care services if they are deemed ‘too violent’ to remain in mainstream general practice and ‘too complex’ to be referred to the SAS.

Addressing these concerns remains a challenge given the dearth of evidence about the implications of removing patients for those affected. Our recent scoping review of the literature on patient removals in the UK found extremely limited evidence on the topic, with the most recent article published over a decade ago. Crucially, we found no published evidence on the delivery or experiences of care post-removal.^7^ The voices of patients on the SAS remain conspicuously absent. We have been addressing some of these gaps through the ongoing SEARCH (Social and Ethical Aspects of Remote and Hybrid Care in the Special Allocation Scheme) study.^7^

## After exclusion

Early findings from the SEARCH study suggest that questions remain about how to adequately meet the needs of patients once the decision has been made to transition them from local GP care to the SAS. Two key issues are emerging here. First, because SAS services cover large geographical areas (sometimes only one per integrated care board), referrals almost always place patients ‘out of area’. This poses serious challenges to providing continuity of care and regular in-person consultations. Patients can find it hard to use public transport, often do not have access to a vehicle, and may be reluctant to access care in unfamiliar or distant locations. Remote care (primarily via telephone) plays an important role in facilitating booking, triage, and consultations, despite the fact we know it is not suitable for every population.^8^ Research into primary care during the COVID-19 pandemic showed, for example, how clinical relationships with patients experiencing homelessness were affected by the rapid digitalisation of care^9^ and how remote consulting can present challenges to identifying and managing safeguarding concerns,^10^ as well as creating unique barriers to access for certain patient groups.^11^

The second issue is that removal from a local GP surgery can make patients ineligible for local services such as community mental health teams. This means that, in practice, SAS providers are forced to provide additional care (beyond the scheme’s intended function) and/or navigate complex alternative routes to mental health, social care, and hospital services. This puts undue burden on those providing SAS services, and could have long-term implications for the provision of sufficient, continuous, and holistic primary care support to patients with SMD and associated complex needs.^5^

## Pathways to inclusion

National and regional stakeholders tell us the intended function of the SAS is to be rehabilitative rather than punitive, and that it is a temporary measure leading to reintegration into mainstream primary care. However, we are seeing that this is far from straightforward to translate into practice, given the structures of the SAS we have described above. For patients, it can be hard to see referral as anything but punishment, especially when they are first referred and are confronted with the distance, loss of local health and social care services, and potential stigma associated with the scheme. We are seeing that SAS providers can and do work hard to mitigate these issues, re-build trust, and make up for the loss of local wraparound services. However, achieving this means that some patients can be reluctant to leave SAS practices once their time on the scheme ends. The added loss of continuity and potential challenges of re-registering at a mainstream GP after a year or more on the SAS mean that reintegration cannot be an afterthought.

## Moving forward

To fully address the complexity of removing and reintegrating patients, we believe it may be helpful to look to existing models of *inclusion health* ^12^ rather than ‘reinventing the wheel’ when it comes to re-thinking *exclusion* in primary care. Applying inclusion health principles in collaboration with those affected could address some of the issues we have raised in this piece by: 1) prioritising accessible, trauma-informed care, with necessary adaptations for digital and remote services; 2) ensuring patients on the SAS are not excluded from other protective within-area services; and 3) supporting providers to ensure patients have clear, safe pathways back to mainstream care. More broadly, we need to address the consistent erosion of general practice funding, which creates a caustic environment in which it is unsurprising that patients may behave in a manner worthy of SAS referral.

Current concerns in the media and the changes to the Primary Medical Services Policy and Guidance appear to be focused on avoiding referrals to a scheme that is struggling to meet the needs of patients. Instead, we need a shift in focus towards violence prevention and creating a primary care context that is inclusive, trauma-informed, and sensitive to the effects of severe and multiple disadvantage.
